# Mitochondrial dysfunction in neurological disorders: Exploring mitochondrial transplantation

**DOI:** 10.1038/s41536-020-00107-x

**Published:** 2020-11-23

**Authors:** Pedro Norat, Sauson Soldozy, Jennifer D. Sokolowski, Catherine M. Gorick, Jeyan S. Kumar, Youngrok Chae, Kaan Yağmurlu, Francesco Prada, Melanie Walker, Michael R. Levitt, Richard J. Price, Petr Tvrdik, M. Yashar S. Kalani

**Affiliations:** 1 Department of Neurological Surgery, University of Virginia Health System, Charlottesville, VA USA; 2 Department of Biomedical Engineering, University of Virginia School of Medicine, Charlottesville, VA USA; 3 Department of Neurosurgery, University of Washington School of Medicine, Seattle, WA USA; 4 Department of Neuroscience, University of Virginia School of Medicine, Charlottesville, VA USA; 5 Present Address: Acoustic Neuroimaging and Therapy Laboratory, Fondazione IRCCS Istituto Neurologico Carlo Besta, Milan, Italy; 6 Present Address: Focused Ultrasound Foundation, Charlottesville, Virginia USA; 7 Present Address: St. John’s Neuroscience Institute, Tulsa, OK 74119 USA

**Keywords:** Translational research, Cell death in the nervous system

## Abstract

Mitochondria are fundamental for metabolic homeostasis in all multicellular eukaryotes. In the nervous system, mitochondria-generated adenosine triphosphate (ATP) is required to establish appropriate electrochemical gradients and reliable synaptic transmission. Notably, several mitochondrial defects have been identified in central nervous system disorders. Membrane leakage and electrolyte imbalances, pro-apoptotic pathway activation, and mitophagy are among the mechanisms implicated in the pathogenesis of neurodegenerative diseases, such as Alzheimer’s, Parkinson’s, and Huntington’s disease, as well as ischemic stroke. In this review, we summarize mitochondrial pathways that contribute to disease progression. Further, we discuss pathological states that damaged mitochondria impose on normal nervous system processes and explore new therapeutic approaches to mitochondrial diseases.

## Introduction

Mitochondria are double-membrane organelles with structurally and functionally distinct outer and inner membranes, separating the intermembrane space from the matrix^1^. The size, shape, and number of mitochondria can vary widely depending on cellular function and environmental stress^2^. The inner mitochondrial membrane harbors the complexes of the respiratory chain and oxidative phosphorylation^1^, key components for cellular bioenergetics and survival. In neurons, mitochondria play additional important roles in calcium homeostasis, control of membrane excitability and neurotransmission and plasticity^3^. Mitochondria possess their own transcriptional and translational machineries required for expression of mitochondrial DNA (mtDNA)-encoded genes^1^. The complex biogenesis makes these organelles particularly vulnerable to accumulating damage during the lifespan of a cell. Sustained mitochondrial damage results in the dysfunction of energy metabolism; accordingly, this leads to decreased ATP production, increased reactive oxygen species (ROS) burden, and reduced calcium buffering, all of which lead to neuronal loss characteristic of both acute and chronic degenerative neurological disorders. Here, we review mitochondrial dysfunction in several major nervous system disorders and traumatic brain injury (TBI). Finally, we discuss new experimental strategies for mitochondrial transplantation into the central nervous system.

### Mitochondrial pathways of disease

**Fig. 1:**
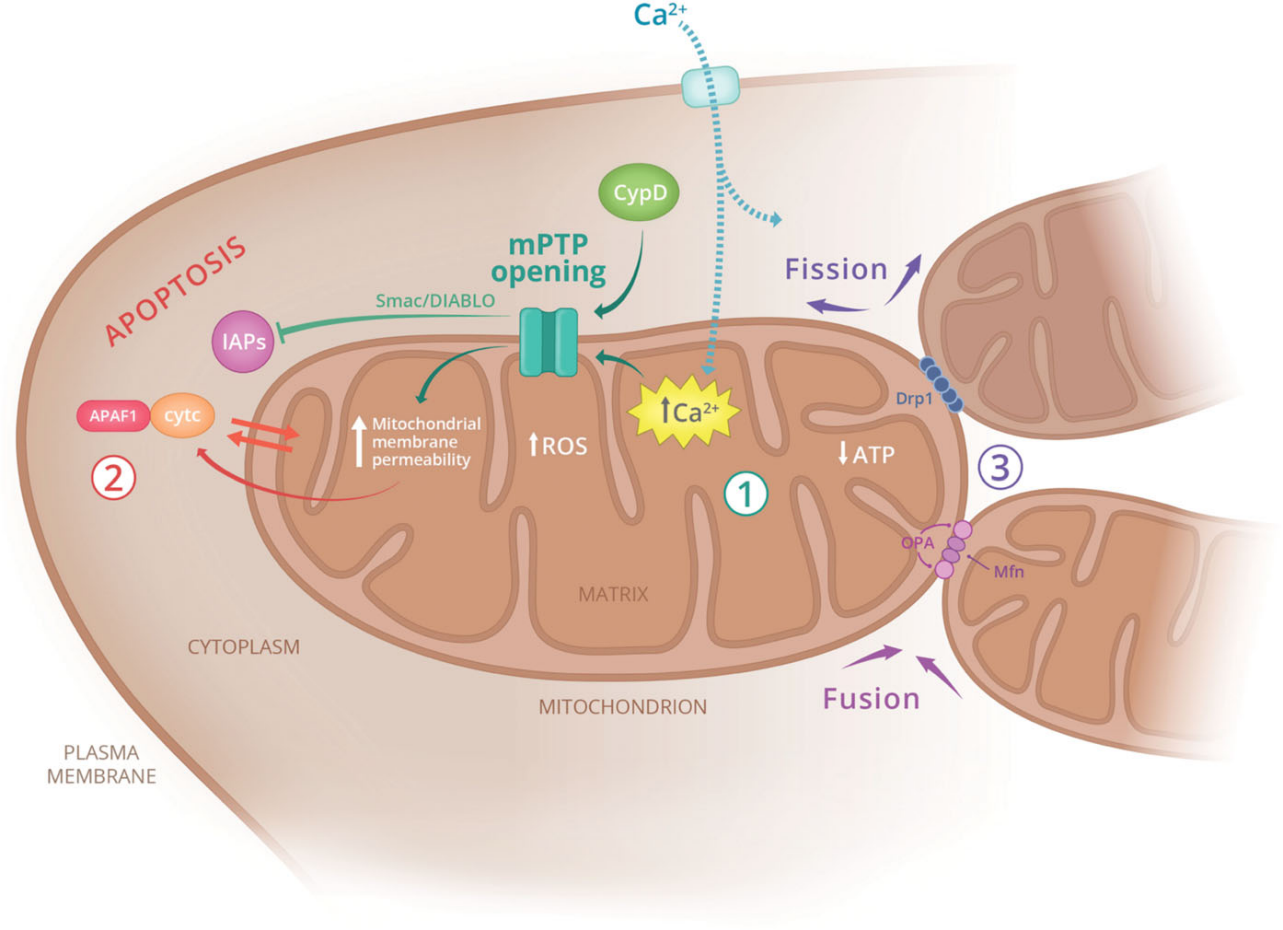
Mitochondrial damage contributes to a wide range of pathologies. The main pathways leading to mitochondria-associated cellular dysfunction include (1) calcium overload in the matrix and mPTP pore opening, (2) cytochrome c release and activation of apoptosis, and (3) defects in mitochondrial fission and fusion affecting mitochondrial longevity. APAF1 apoptotic protease activating factor 1, ATP adenosine triphosphate, CypD cyclophilin D, cytc cytochrome c, Drp1 dynamin-related protein 1, IAPs inhibitor of apoptosis proteins, Mfn mitofusin, mPTP mitochondrial permeability transition pore, OPA optic atrophy mitochondrial fusion protein, ROS reactive oxygen species, Smac/DIABLO pro-apoptotic mitochondrial protein.

Disruption of normal mitochondrial function is detrimental to cell viability. Neurons are particularly dependent on mitochondria for calcium buffering and ATP production and, therefore, are highly susceptible to mitochondrial defects^4^. During metabolic distress, multiple pathological pathways are activated in mitochondria, including opening of the mitochondria permeability transition pore (mPTP), leakage of cytochrome c into the cytoplasm, induction of programmed cell death, and mitophagy (Fig. 1).

In response to cell damage, excessive Ca^2+^ accumulation occurs in the matrix and serves as the principal trigger for opening of the mPTP, which acts as a high-conductance channel^5^. The molecular identity of this pore has yet to be determined, but cyclophilin D (CypD) is known to facilitate mPTP activation under physiologic conditions^6^. Opening of the mPTP results in an altered proton electrochemical gradient, as well as compromised membrane potential and changes in pH gradient. Increased mitochondrial membrane permeability allows for the accumulation of ROS, resulting in greater oxidative stress, causing a self-propagating chain reaction of cell membrane lipid peroxidation, destruction of membrane lipids and significant tissue damage^7^. This results in an overall reduction of ATP generation that ultimately leads to the activation of the intrinsic apoptosis pathway and cell death.

Beyond apoptosis, there are several other forms of programmed cell death, including necroptosis, parthanatos, pyroptosis, and ferroptosis, all of which have been tentatively linked to mitochondria, but whether mitochondrial disruption is upstream or downstream is under debate^8–12^. Ferroptosis is of particular interest in relation to neurological disorders as it is a major mechanism for cell death associated with ischemic organ injury and brain damage and has been implicated in neurodegeneration^13–16^. Ferroptosis does not lead to the stereotypical morphological changes seen in cells undergoing apoptosis or necrosis; instead it is predominantly characterized by shrinkage of mitochondria, with increased membrane density and disruption of the cristae^17^. Interestingly, many of the drugs influencing ferroptosis appear to be targeted to mitochondria^9,18,19^. Ferroptosis is initiated when the iron-dependent accumulation of toxic lipid hydroperoxides (lipid ROS) outbalances a cell’s ability to compensate with cellular redox homeostasis. To repair the membrane damage caused by lipid ROS, the lipid hydroperoxidase glutathione peroxidase 4 (GPX4) converts lipid ROS to non-toxic lipid alcohols^20^. However, with increased iron accumulation and ROS production, or depletion of glutathione and decreased activity of GPX4, lipid ROS accumulate; this leads to ferroptosis due to membrane disorganization and non-specific membrane perforation caused by the toxic lipids^21^.

The increase in mitochondrial permeability also mediates leakage of proteins into the cytoplasm, including the protein cytochrome c^6^. Cytochrome c is involved in normal mitochondrial metabolism and serves as an electron carrier from complex III to complex IV in the electron transport chain (ETC). When released into the cytoplasm, cytochrome c binds a protein called apoptosis-activating factor-1 (APAF-1), forming a multimeric structure called the apoptosome, which triggers the caspase cascade. The cascade pathway activates executioner caspases, such as caspase-3, -6, and -7, which promote enzymatic cleavage of DNA and nuclear fragmentation associated with apoptosis^22^.

Mitochondrial fission and fusion have been recognized as critical processes in mitochondrial and cellular health. Both processes are mediated by guanosine triphosphatases (GTPases)^23^. Fusion mediates a process known as “functional complementation,” whereby functional components from a healthy mitochondrion are able to compensate for dysfunctional components of the damaged organelle. Fission plays a major role in facilitating mitochondrial autophagy, otherwise referred to as mitophagy. Large, elongated mitochondria are less amenable to mitophagy. Fission is able to facilitate autophagosomal encapsulation by both reducing mitochondrial size and segregating dysfunctional fractions of mitochondrial networks^24^.

### Alzheimer’s disease (AD)

AD is a chronic, neurodegenerative condition recognized as the most common cause of dementia. Patients with AD experience a wide array of symptoms ranging from cognitive decline to behavioral impairment, with symptoms worsening over time. AD is characterized by two pathological phenomena in the brain: aggregations of the amyloid beta peptide (Aβ) and phosphorylated tau protein tangles (NFTs)^25^.

Although several hypotheses have been proposed, the mechanism of AD pathology remains unclear. The mitochondrial cascade hypothesis suggests that alterations of the mitochondria-associated endoplasmic reticulum membranes (MAM) and the mPTP are key elements in AD pathology^26^. Combined with environmental and lifestyle factors, once a certain threshold of mitochondrial dysfunction is met, this triggers Aβ aggregation, tau phosphorylation, and synaptic loss and degeneration^27^.

Mitochondrial permeability transition is an abrupt increase in the permeability of the inner mitochondrial membranes to solutes of <1500 Da. The mitochondrial permeability transition is caused by the opening of the mPTP. The opening of the mPTP, facilitated by CypD, ultimately causes osmotic swelling of the mitochondrial matrix and a rupture of the outer mitochondrial membrane. CypD has been shown to be significantly increased in Aβ-containing mitochondria within the temporal lobe and hippocampus of AD patients^28^. Additionally, there was increased CypD translocation to the IMM, increased ROS production, and decreased calcium buffering capacity, indicating a strong interaction of CypD with Aβ in AD brains^29^. The interaction of CypD with Aβ serves as a trigger for the formation of mPTP. This suggests an abnormal accumulation of amyloid influences the efficiency of the mitochondrial oxidative phosphorylation process, causing decreased ATP production and increased ROS production^28,29^.

Blocking the formation of the mPTP by genetically depleting CypD or using a CypD inhibitor may prevent the detrimental effects of AD. For instance, genetic depletion of CypD has been shown to reduce mitochondrial swelling, oxidative stress, and apoptosis in mouse models^28^. CypD-deficient mice overexpressing APP and Aβ (APP mice) were able to better regulate their mitochondrial functions including mitochondrial complex IV activity and ATP production^30^. Aged AD mice showed similar improvements as well, confirming that CypD depletion has the potential to be a therapeutic strategy for ameliorating mitochondrial impairment caused by AD^28^.

### Parkinson’s disease (PD)

PD is the most common movement disorder and the second most prevalent neurodegenerative disease worldwide^31^. This presently incurable illness is characterized by progressive degeneration and death of dopaminergic neurons. It leads to decreased dopamine levels and expression of intracytoplasmic inclusions containing fibrillar α-synuclein, or Lewy Bodies, within the surviving neurons of the substantia nigra. The loss of dopamine causes dysregulation of the basal ganglia circuitry, which results in the classic triad of slowness of movement (bradykinesia), rigidity, and resting tremor. Given that mitochondria are key for energy metabolism, calcium homeostasis, and control of membrane excitability, mitochondrial dysfunction is suspected to be a trigger in the pathology of PD.

Accordingly, deficiency in complex I of the ETC have been described in PD patients^31^. Decrease of complex I activity was found in the platelets and skeletal muscle and subsequent post-mortem analyses confirmed complex I deficiency in the substantia nigra of the affected individuals^32^. In experiments where mtDNA derived from PD patients was transfected into normal cells and mitochondrial function have been evaluated, the recipient cells had reduced complex I activity, lower mitochondrial membrane potential and altered calcium homeostasis^33^.

These studies implicate mtDNA in neuronal degeneration observed in PD. To further address this question, Ekstrand et al.^34^ conditionally deleted the gene encoding mitochondrial transcription factor A (*Tfam*) in dopaminergic neurons. In situ hybridization showed reduced expression of mtDNA-encoded cytochrome-c subunit I mRNA and histochemical analysis confirmed markedly reduced cytochrome-c enzyme activity in these neurons. Remarkably, these mice displayed progressive tremor, twitching, and limb rigidity by 20 weeks of age. Intraneuronal inclusions were also observed; however, these inclusions did not contain α-synuclein^34^.

Recently, the PINK1/Parkin pathway has been identified as the most frequent cause of early-onset PD^35^. Decrease in mitochondrial membrane potential stabilizes PINK1 (PTEN-induced kinase 1) on the outer mitochondrial membrane. PINK1 phosphorylates ubiquitin at this site, which results in the recruitment of Parkin, an E3 ubiquitin ligase. Parkin is then also phosphorylated by PINK1, leading to its activation, formation of the autophagosome, and subsequently lysosome-mediated degradation of the mitochondria. Genetic mutations in the *Parkin* gene are associated with ~50% of familial PD, and are the most common cause of PD in people younger than 20 years. Patients with *Parkin* mutations present with symmetrical parkinsonism, slow progression, and good response to levodopa^36^.

Mutations in PINK1 have also been reported as a cause of autosomal recessive PD, particularly in patients with early onset parkinsonism prior to 40 years of age^37^. Patients with this mutation have a good response to levodopa and a slow progression. Autopsy of patients’ brains with PINK1 mutation show substantia nigra cell loss and Lewy body formation^37^. Conversely, over-expression of PINK1 restores normal mitochondria morphology and protects from ROS^38^.

Unexpectedly, several clinical reports document beneficial effects of deep brain stimulation (DBS) on mitochondrial volume. DBS of the subthalamic nucleus is an effective surgical treatment in patients with advanced PD, and it is thought to reduce nigral glutamate excitotoxicity by inhibiting the subthalamic nucleus. Recently, Mallach et al. performed post-mortem analysis of striatal dopaminergic structures and found that mitochondrial volume was significantly higher in the brains that received DBS, with levels similar to those in controls. The authors attribute these results to increased mitochondrial biogenesis, decreased mitochondrial fission, or increased fusion^39^. Although merely correlative at the present time, these findings provide further encouragement for alternative therapies using mitochondrial transplantation.

### Huntington’s disease (HD)

HD is a neurodegenerative disorder caused by a pathological expansion of CAG repeats within the huntingtin gene, leading to polyglutamine expansion in the encoded protein. Degeneration occurs initially in the caudate and striatum and progresses to involve the cerebral cortex. Clinical features of HD include progressive motor dysfunction and dementia. The underlying mechanisms leading to pathology have not been determined, and the pathogenesis of the disease may be multifactorial.

Much work has been done to show that mutant huntingtin leads to transcriptional dysregulation and pathogenesis^40^. Notably, huntingtin has also been shown to interfere with a transcription factor that modulates mitochondrial gene expression, PGC-1α. This provides a link by which mutant huntingtin effects both transcriptional regulation and mitochondrial function. PGC-1α is a transcriptional co-activator that interacts with transcription factors that regulate the expression of mitochondrial respiratory genes including NRF-1 and NRF-2, which play a role in mitochondrial function and energy homeostasis. Recent studies have provided evidence that the expression of PGC-1α is repressed by mutant huntingtin and PGC-1α knockout mice have defects in energy metabolism and exhibit spongiform degenerative changes in the striatum^41^. Interestingly, the striatum appears to be especially sensitive to disruptions in mitochondrial function. Striatal mitochondria contain more CypD than cortical mitochondria and are more sensitive to calcium-induced mPTP opening, which may contribute to the regional differences in timing and severity of degeneration in HD^42^.

There are multiple lines of evidence showing that mitochondria are abnormal in HD patients and in genetic models of HD. The mutant form of huntingtin leads to compromised mitochondrial morphology, energy metabolism, and increased oxidative damage, as well as calcium-handling deficits^42,43^. Descriptive ultrastructural studies showed abnormal mitochondria morphology in patients with HD^44^, and further work in mice and humans showed that mutant huntingtin triggers mitochondrial changes, including fragmentation, through abnormal interactions with the mitochondrial fission GTPase, dynamin-related protein-1 (DRP1)^45^. Mutant huntingtin stimulates enzymatic activity of DRP1, leading to mitochondrial fragmentation, defects in anterograde and retrograde mitochondrial transport, and neuronal cell death; notably, rescue of these defects is achieved by inhibiting DRP1 GTPase activity^45^. Mitochondrial function is also abnormal in HD. Mitochondria from huntingtin knock-in rodents display an increased sensitivity to calcium-induced changes in respiration and mPTP opening^42^. Several studies show that addressing mitochondrial dysfunction may protect striatal neurons from mutant huntingtin-induced toxicity. For example, PGC-1α overexpression in models of HD corrects some deficits induced by mutant huntingtin^46^.

### Ischemic stroke

Ischemic stroke is one of the leading cause of morbidity and the second leading cause of death worldwide^47^. Due to its high intrinsic metabolic activity, the brain is sensitive to prolonged reduction in oxygen and glucose supply that typically result from arterial thrombosis or embolism. Victims of ischemic stroke often experience paralysis, impaired speech, or loss of vision. Because of the narrow window of therapeutic opportunity, a low number of stroke patients receive appropriate medical intervention in time, and not all patients are eligible for thrombolysis. Also, after reperfusion, some patients may experience a reperfusion injury, caused by recanalization of the blood flow that leads to leukocyte infiltration, platelet and complement activation, post-ischemic hyper-perfusion, and breakdown of the blood–brain barrier. Therefore, it is imperative to develop new treatment strategies.

Within minutes after arterial occlusion, the ischemic brain begins depolarizing mitochondrial membranes, which leads to depletion of ATP production, overproduction of ROS and accumulation of PINK1 and unfolded protein response (UPR). The reduction of ATP triggers ischemic cascades such as membrane ion pump failure, plasma membrane depolarization and efflux of cellular potassium, influx of sodium, calcium, chloride, and water^48^. All of these factors lead to opening of mPTP, liberation of cytochrome c, activation of caspase 3 and consequently, execution of apoptotic death^49^.

In ischemic brain injury, mitochondrial fission promotes neuronal death while mitochondrial fusion allows damaged mitochondria to be repaired^50^. Drp1, Fis1, and Endophilin B1 proteins are required for mitochondrial fission, but they can also affect the regulatory processes that control mitochondria fusion^51^. Conversely, Opa1, Mfn1, and Mfn2 are mitochondrial fusion proteins that have also been identified in mitochondrial fission^52^. Using mouse models of ischemic brain injury, studies have shown that changes in expression levels of Drp1, Fis1, Opa1, and Mfn2 can inhibit or promote fusion and fission^50^. Another promising approach is focused on the process of mitophagy. Studies have suggested that mitophagy enables neurons to remove damaged mitochondria, prevents apoptotic cascade activation and, consequently, allows neurons to survive after ischemic stress^53^.

## Mitochondrial transplantation as a therapeutic approach

Irreversible injury to mitochondria is a cornerstone of pathogenesis of neurological diseases. Therefore, given the critical role of these organelles in disease onset and progression, strategies aimed at mitochondrial transplantation have recently gained interest.

### Lessons from cardiac disease

Mitochondrial transplantation as a therapeutic approach for cardiovascular diseases has been pioneered by McCully et al.^54–56^. In their first study, the authors isolated mitochondria from autologously harvested muscle tissue. After assessing mitochondria viability, the authors directly injected the isolated mitochondria into the ischemic zone of a rabbit’s heart, just prior to reperfusion. The authors demonstrated improved global and regional post-ischemic myocardial functional recovery and significantly enhanced cellular viability in the ischemic zone^55^. Masuzawa et al.^57^ also demonstrated that transplantation of mitochondria increases the total tissue ATP content, augmenting expression of mitochondrial proteins and precursor metabolites that support energy production and cellular respiration. In addition, improved vascularization, protection against apoptosis, and enhanced cardiac functional recovery was observed^57^.

In other studies by McCully et al. the authors transplanted exogenous mitochondria through the coronary vasculature to increase the therapeutic potential, demonstrating that mitochondria can be delivered into the ischemic heart intravascularly with similar outcomes as direct injection^54,56^. The advantage of the intravascular approach when compared with direct injection is that intra-arterial injection permitted higher concentrations of mitochondria to be delivered to ischemic areas of the heart and with better distribution such that individual mitochondria were observed within capillaries, cardiomyocytes, and interstitial spaces. In contrast, direct injection concentrates the mitochondria to that particular region. Their studies have also demonstrated that once transplanted, exogenous mitochondria provided cardioprotection both extracellularly and intracellularly. Non-viable mitochondria, mitochondrial fractions, mtDNA or RNA, and exogenous ATP or ADP did not confer cardioprotection when administered into the ischemic heart.

The first study to apply mitochondrial transplantation in humans was by Emani et al.^58^. Pediatric patients who sustained a myocardial ischemic event following cardiac surgery that was not ameliorated by surgical intervention and extracorporeal membrane oxygenation (ECMO) support were eligible for autotransplantation. Mitochondria were isolated from accessible sources of skeletal muscle following sternotomy, such as the rectus abdominis, sternohyoid, pectoralis, or sternocleidomastoid muscles^59^. Ten 100-μL injections containing 1 × 10^7^ ± 1×10^4^ mitochondria each were delivered by direct injection using a 28-gauge needle to the myocardium affected by ischemia-reperfusion. No adverse short-term complications related to mitochondrial injection, including arrhythmia, intra-myocardial hematoma, or scarring was experienced by any of the patients. Of the five patients, four had improved ventricular function and were removed from ECMO support^59^.

### Application to neurological disorders

Zhanq and others have performed muscle-derived autologous mitochondrial transplantation into the lateral ventricles of the rodent brain^60^. They used a model of middle cerebral artery (MCA)-occlusion and replenished damaged mitochondria after ischemic stroke with exogenous mitochondria. They showed that intraventricular transplantation of mitochondria reduced cellular oxidative stress and apoptosis, decreased brain infarct volume, reversed neurological deficits, attenuated reactive astrogliosis, and promoted neurogenesis. They noted that a higher number of viable mitochondria in the cerebrospinal fluid correlated with better neurological outcomes^60^.

A new promising therapy for stroke involves the transfer of mitochondria into the affected brain region because mitochondria may activate signals for cell survival^61^. In 1983, the existence of vesicle release from cells was reported, but their function with respect to cellular communication was described more recently^62^. Vesicles are secreted by various cell types, for example, oligodendrocytes^63,64^, and can contain signaling molecules and small organelles such as mitochondria. There is evidence to suggest that mitochondria can be released in extracellular vesicles in response to inflammatory stimuli^65^. Recently, mitochondria have been shown to transfer from astrocytes to neurons after an ischemic event^66^, suggesting that astrocytes may release mitochondrial particles extracellularly and they may enter neurons to support cell viability and recovery after stroke. In addition, it is possible that transfer can occur through direct cellular connections. The study that introduced the possibility of intercellular transfer of mitochondria described nanotubular structures formed de novo between cells that create complex networks, capable of cell-to-cell organelle transfer^67^.

Another possible source of mitochondria is from endothelial progenitor cells (EPCs), which are immature endothelial cells present in the blood stream. They provide neuroprotection against ischemic strokes by playing an important role in restoring disrupted blood–brain barrier. In 2018, using an in vitro stroke model, Hayakawa et al.^68^ observed the presence of functional extracellular mitochondria with high membrane potentials released by EPCs, providing evidence that EPCs can release mitochondria. Furthermore, the authors showed that EPC-derived extracellular mitochondria can be transferred to the brain endothelial cells and restore mitochondrial functions, reducing endothelial permeability and increasing cell viability^69^. Finally, the authors observed that the mtDNA of transferred mitochondria upregulated genes that promoted angiogenesis and blood–brain barrier function and concluded that the transferred mitochondria can reverse altered endothelial cells’ functions^69^.

Indeed, isolation of biochemically active mitochondria can be readily performed in the clinical setting^70^. The strengths of this approach include rapid isolation protocol (30 min), autologous tissue of the patient from a simple needle biopsy, and the viability and high yield of isolated mitochondria. These features make mitochondria a promising candidate in clinical trials for a wide array of neurological disorders.

### Strategy for mitochondrial delivery and tracing in the central nervous system

**Fig. 2:**
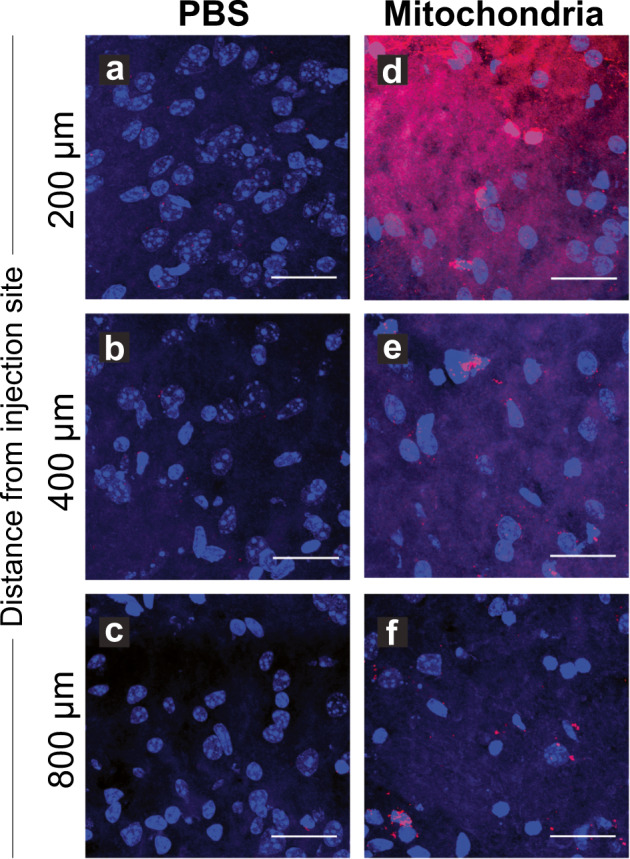
Stereotactic injection facilitates efficient delivery of mitochondria into the brain parenchyma. Injected brains were harvested, cryo-sectioned and stained with nuclear stain DAPI (blue). **a**–**c** Control brain injected with PBS, **d**–**f** Experimental brain injected with exogenous mitochondria labeled with MitoTracker Red CMXRos (Invitrogen). Both mice were injected directly into the striatum and striatal sections were imaged with confocal microscopy. The images were acquired at various distances from the injection site: **a**, **d** 200 μm, **b**, **e** 400 μm, and **c**, **f** 800 μm. Mitochondria were found to diffuse in the parenchyma and detected over 1 mm from the injection site. Scale bar, 25 μm.

**Fig. 3:**
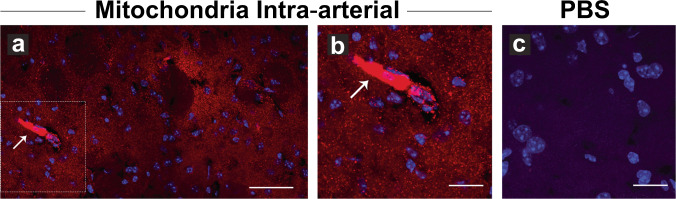
Intra-arterial injection can deliver mitochondria in the ischemic brain parenchyma. Mice were stroked with middle cerebral artery occlusion, injected intraarterially and their brains were cryo-sectioned and stained with DAPI (blue). **a** and **b** Stroked brain injected with exogenous mitochondria pre-labeled with MitoTracker (red), **c** Stroked brain injected with PBS. Both animals were injected in the common carotid artery following middle cerebral artery occlusion. Images were acquired in the striatum with confocal microscopy. Mitochondria crossed the blood–brain barrier and localized throughout the parenchyma. Occasionally, mitochondria were found deposited in the blood vessels (white arrow). **b** Higher magnification of a mitochondrial deposit shown in **a**. **a** Scale bar, 50 μm. **b**, **c** Scale bar, 25 μm.

Mitochondria can be delivered to the central nervous system using intraparenchymal/ intraventricular or intra-arterial injection. Stereotactic intracerebral injection of mitochondria allows for deposition of the mitochondria within the parenchyma. Mitochondria diffuse from the initial site of injection, even though the spread remains limited (Fig. 2). The intra-arterial administration of mitochondria has emerged as an alternative route of delivery. Although some mitochondria are retained within the blood vessels, a substantial amount of organelles traverse the blood–brain barrier after blood–brain barrier disruption due to ischemia-reperfusion injury, and occasionally incorporate into neurons and other cells of the central nervous system (Fig. 3).

As evident from these experiments, tracing the transplanted mitochondria and interpreting the outcomes requires staining with appropriate dyes. The synthetic MitoTracker Red CMXRos dye has been widely used to assess efficiency in transplantation studies^60,71^. This technique has been employed in the previously mentioned study documenting the transfer of mitochondria between astrocytes^66^, for visualization of intravenously administrated mitochondria for treating experimental PD^72^, or for documenting transfer of isolated astrocytic mitochondria into glioma cells in vitro and in vivo^73^. Different color MitoTracker dyes, such as Mitotracker Green FM and Mitotracker Deep Red FM, can be used to label mitochondria in different cell populations separately and study mitochondrial traffic between the two cell types^74^. Conveniently, the MitoTracker dyes can be efficiently fixed in histological preparations (Figs. 2 and3). An alternative approach to detecting membrane potential in isolated mitochondria are ratiometric fluorescent dyes, such as JC-1. This probe undergoes potential-dependent accumulation in mitochondria, indicated by a fluorescence emission shift from the green monomer at lower dye concentrations to red aggregates which are formed as the dye concentration increases. This probe afforded evidence that extracellular dysfunctional mitochondria propagate injury from microglia to astrocytes to neurons, whereas extracellular functional mitochondria are neuroprotective^60,66,72,73,75–77^.

However, synthetic dyes also have many disadvantages; including potential mitochondrial toxicity following membrane loading with fluorescent probes and dye leakage out of mitochondria when used at higher concentrations. Therefore, endogenous labeling with genetically encoded indicators can be a more precise tool for investigating the frequency and magnitude of mitochondrial transfer with less cytotoxicity. DNA constructs are used to drive expression of fluorescent proteins into mitochondria in a cell-specific fashion. The targeting pre-sequence of subunit VIII of human cytochrome c oxidase (COX8) proved a reliable method for chimeric protein delivery to the mitochondrial matrix in various expression systems. After entering the matrix, the pre-sequence is cleaved, liberating the linked polypeptide; soluble fusion proteins such as GFP then undergo rapid diffusion in the mitochondrial matrix^78^. Green and red fluorescent protein variants have been successfully targeted to mitochondrial matrix, facilitating mitochondrial transplantation studies^79–82^. A special mention in this regard goes to the genetically encoded sensor that reports physiologic properties of the mitochondrial matrix, the MitoTimer reporter. It is a variant of the red fluorescent protein, DsRed1, which undergoes fluorescence shifts over time from green to red as the protein matures. This molecular clock gives temporal and spatial information on protein turnover, providing insight in fundamental processes such as mitophagy^83,84^.

Using synthetically and genetically labeled mitochondria in two different transplantation strategies, we have found that both modes of delivery are technically feasible, but intra-arterial injection results in a wider distribution of mitochondria in the parenchyma, at least in the context of blood–brain barrier disruption after ischemia-reperfusion injury. In contrast, stereotactic injections show not only limited distribution around the injection site, but also the potential for additional complications due to the more invasive nature of this approach, including hemorrhage or infection at the site of injection. Therefore, intra-arterial delivery of mitochondria should be a superior approach for future clinical applications. Furthermore, our ongoing research suggests that judicious application of blood–brain barrier permeating technologies such as focused ultrasound might further improve the distribution of transplanted mitochondria in the ischemic penumbra and reduce the formation of mitochondrial deposits in blood vessels.

### Therapeutic mechanisms of transplanted mitochondria

The exact mechanisms by which transplanted mitochondria exert their therapeutic effects remain unclear. One school of thought contends that the benefits of mitochondrial transplantation are imparted by direct mitochondrial entry into neurons. Using a mouse model of ischemic stroke, Hayakawa and collaborators suggest an integrin-mediated Src/Syk signaling pathway to be involved in mitochondrial transfer from astrocytes into neurons^66^. Studies looking at pulmonary epithelial cells implicate gap junction formation via CX43 connexins and tunneling nanotubes as necessary to mediate both attachment and movement of mitochondria, with dynamin/clathrin or integrin-regulated endocytosis being necessary for engulfment^85–87^. Other mechanisms may involve cell fusion, microvesicles, or direct mitochondrial uptake^4,88^.

Evidence for internalization of exogenous mitochondria relies on high-resolution imaging and 3D reconstruction in brain sections after mitochondrial transplantations^66^. As previously discussed, genetically encoded markers such as tGFP or mitodsred afford superior technique for determining intracellular localization^79^. Using this strategy, Rocca and colleagues detected mitochondrial transfer in vivo from hematopoietic stem cell-derived microglia and macrophages into neurons in the mouse model of Friedreich’s ataxia^82^. Conversely, in the rat model of spinal cord injury, transplanted tGFP-labeled mitochondria co-localized with multiple resident cell types in the spinal cord, but they were notably absent in neurons^80^. It is presently unclear how these contradictory findings can be reconciled, and new research will hopefully shed light on this important issue. Alternative methods may also be needed to clarify the processes. For example, mitochondria isolated from cells pre-incubated with BrdU allow an alternative tracing method for transplanted organelles. Several weeks after mitochondrial transplantation in the striatum in a rat model of focal cerebral ischemia, confocal microscopy analysis revealed that BrdU signals were detected in neurons, astrocytes, and microglia in the peri-infarct area^89^. However, the authors also concluded that the low efficacy of mitochondrial internalization could not completely account for the high rate of rescue in this study^89^.

It is becoming apparent that mitochondria may also exert their beneficial effects outside of the cell in the interstitial space, and measurements of ATP, JC-1 fluorescence ratio, and oxygen consumption suggest they can retain activity extracellularly^90^. Free mitochondria may support increased survival by supplying ATP or scavenging ROS^89^. Morphologically dynamic, mitochondria have the ability to form mitochondrial networks as instructed by the surrounding metabolic state^91^. It is possible that transplanted mitochondria may function to initiate cell signaling cascades and salvage metabolic derangement via these networks, acting as a signaling organelle^92,93^. Cellular and extracellular mitochondria cross-talk may be mediated by nanotubular cell-to-cell connections, which are predicted to transfer charges and electromagnetic radiation between cells^94^. These novel findings and hypotheses are intriguing, and more work is needed to better delineate the molecular mechanisms by which internalized or extracellular mitochondria impart their therapeutic roles in the central nervous system.

## Conclusions

Viable mitochondria are critical for cellular function and homeostasis, and disruption of mitochondrial function contributes to the pathogenesis of a variety of neurological conditions. Mitochondrial therapy may have a place in the treatment of a diverse array of disorders, including neurodegenerative diseases, ischemic stroke, and TBI. Transplantation of mitochondria is likely to offer a means to mitigate damage to diseased or injured brain and refinement of techniques for delivery warrants further study.

## Methods

### Animals

Male and female C57BL/6J mice were purchased from the Jackson Laboratory (Bar Harbor, Maine, USA). Only adult animals (8–10 weeks) were used in this study. All mice were housed in a controlled environment on 12 h light/dark cycles and fed a standard chow. All experiments were approved by the Institutional Animal Care and Use Committee of the University of Virginia.

### Mitochondrial isolation

The gastrocnemius muscle was dissected, and 2 cm of muscle tissue was placed in 1× PBS. The tissue was transferred to a dissociation C tube (130-093-237, Miltenyi Biotec) containing 5 ml of cold homogenizing buffer (HB) (300 mM sucrose, 10 mM HEPES, and 1 mM EGTA, pH 7.4), and homogenized using the gentleMACS Octo Dissociator (130-095-937, Miltenyi Biotec) according to the manufacturer’s instructions. The homogenates were placed on ice, mixed with 250 µl of Subtilisin A (9014-01-1, Sigma-Aldrich; 4 mg/ml in HB) and incubated on ice for 10 min. The suspension was then filtered through a 40-µm cell strainer (352340, Corning) into a pre-chilled 50 ml conical tube. Next, 250 µl of bovine serum albumin (BSA, 20 mg/ml in HB) was added to the filtrate, mixed and passed stepwise through 40 and 10-µm cell strainers (43-50010-03, PluriSelect) into another pre-chilled 50 ml conical tube. The filtrate was divided into three pre-chilled 1.5 ml microfuge tubes and centrifuged at 9000×*g* for 10 min at 4 °C. Finally, the supernatant was removed and re-suspended with 1 ml of cold respiration buffer (RE).

### MitoTracker staining

Mitochondrial pellets were re-suspended in 94 µl of freshly prepared MitoTracker Red CMXRos solution (M7512, Invitrogen; 200 nM in DMSO) and incubated at room temperature for 20 min. Next, mitochondria were divided into three 1.5 ml microfuge tubes and washed with 1.5 ml of RE in each tube, in order to remove excess of DMSO. The tubes were spun at 9000×*g* for 10 min at 4 °C. Finally, the pellets of labeled mitochondria were re-suspended with 1 ml of RE.

### Transient proximal middle cerebral artery occlusion (tpMCAo)

Under isoflurane anesthesia, the right-side arteries involved in the blood supply of the brain were exposed, including the common carotid artery (CCA), external carotid artery (ECA), and internal carotid artery (ICA). The ECA was tied with a 6–0 silk suture, then proximal trunk of the CCA was tied, and a microclip (15911, WPI) was placed on the ICA. Next, a small incision was made in the CCA using microscissors and a 6.0 monofilament (6022910PK10, Doccol Corp.) was inserted into the ICA. The filament was advanced until detecting resistance, indicating occlusion of the MCA. The occluding filament in the CCA was temporarily tied to prevent repositioning, the skin was closed and the mouse was allowed to recover in a heated cage. At 1 h, the CCA was re-exposed and the monofilament was removed to induce reperfusion. Finally, the CCA was permanently tied above the craniotomy, the skin was closed with 4–0 suture, treated with buprenorphine and allowed to recover.

### Intra-arterial delivery of mitochondria

One hour after MCA occlusion, the mice were anesthetized and set up for reperfusion as described before. Immediately prior to the withdrawal of the occluding filament, a micro-clip was placed on the ICA artery to avoid retrograde bleeding. The existing arteriotomy in the CCA was then used to insert an intra-arterial catheter (SAI infusion technologies, REF: MAC-01) filled with mitochondria or buffer solution. To immobilize, the catheter was tied in the CCA. Using a connected Hamilton syringe, slow infusion of 200 µl of the fresh mitochondrial suspension was performed. The catheter was then removed from the ICA and the CCA was permanently tied. The skin was closed and analgesics were provided as above.

### Stereotactic delivery of mitochondria

Mice were anaesthetized with isoflurane, the scalp was shaved and the animal was positioned in a stereotaxic frame (Kopf Instruments). Using aseptic techniques, a midline incision was made with small surgical scissors. The skin was removed and bregma and lambda were exposed. A small burr hole was drilled over the target area with a hand-held drill. A 5 µl Hamilton syringe filled with mitochondrial suspension was attached to the stereotaxic apparatus moved to the stereotactic target in the striatum. 2 µl of mitochondria suspension were slowly deposited using Hamilton syringe. The skin was sutured, the animal was treated with analgesics and allowed to recover in a heated cage.

### Histochemistry

Transcardially perfused brains were harvested and placed in buffered 4% formaldehyde for 4 h, cryoprotected with sucrose, snap frozen in OCT and kept at −80 °C until cryo-sectioning. 20 µm-thick coronal sections were mounted on slides (Fisherbrand; 12-550-15), coverslipped with ProLong Gold mounting media with DAPI (Invitrogen; P36935).

### Confocal imaging and analysis

Confocal images were acquired with the Leica TCS SP8 confocal system, equipped with a ×20 0.70 NA, ×40 1.30 NA, and ×63 1.40 NA lenses. All images were acquired at 1024 × 1024-pixel resolution. In order to quantify mitochondria, we used IMARIS software (Bitplane) to analyze images from the penumbra area of brains exposed to different treatments. Using surface rendering, fluorescence thresholding, and masking, we created 3D rendering of the vessels and clusters of mitochondria to quantify the amount of mitochondrial signal in the different compartments. Other quantifications were performed with FIJI software (NIH) and statistical tests were done in GraphPad Prism software.

### Reporting summary

Further information on research design is available in the Nature Research Reporting Summary linked to this article.

## Supplementary information

Reporting Summary Checklist

## Data Availability

All relevant data supporting the findings of this study are available from the corresponding author upon reasonable request.
