# Author Correction: Mitochondrial dysfunction in neurological disorders: exploring mitochondrial transplantation

**DOI:** 10.1038/s41536-021-00125-3

**Published:** 2021-03-02

**Authors:** Pedro Norat, Sauson Soldozy, Jennifer D. Sokolowski, Catherine M. Gorick, Jeyan S. Kumar, Youngrok Chae, Kaan Yağmurlu, Francesco Prada, Melanie Walker, Michael R. Levitt, Richard J. Price, Petr Tvrdik, M. Yashar S. Kalani

**Affiliations:** 1 Department of Neurological Surgery, University of Virginia Health System, Charlottesville, VA USA; 2 Department of Biomedical Engineering, University of Virginia School of Medicine, Charlottesville, VA USA; 3 Department of Neurosurgery, University of Washington School of Medicine, Seattle, WA USA; 4 Department of Neuroscience, University of Virginia School of Medicine, Charlottesville, VA USA; 5 Present Address: Acoustic Neuroimaging and Therapy Laboratory, Fondazione IRCCS Istituto Neurologico Carlo Besta, Milan, Italy; 6 Present Address: Focused Ultrasound Foundation, Charlottesville, Virginia USA; 7 Present Address: St. John’s Neuroscience Institute, Tulsa, OK 74119 USA

**Keywords:** Translational research, Cell death in the nervous system

Correction to: *npj Regenerative Medicine* 10.1038/s41536-020-00107-x, published online 23 November 2020

The original version of this Article contained errors in the author affiliations. The present affiliations of Francesco Prada with Acoustic Neuroimaging and Therapy Laboratory, Fondazione IRCCS Istituto Neurologico Carlo Besta, Milan, Italy; and Focused Ultrasound Foundation, Charlottesville, Virginia, USA were inadvertently omitted. The present affiliation of Yashar Kalani with St. John’s Neuroscience Institute, Tulsa, OK 74119 was inadvertently omitted.

Additionally, the original version of this Article incorrectly omitted acknowledgement of Yashar Kalani as co-corresponding author with the following email address: stemcelldoctor@gmail.com.

These have now been corrected in both the PDF and HTML versions of the Article.

